# Transcatheter aortic valve replacement: impact of pre-procedural FEops HEARTguide assessment on device size selection in borderline annulus size cases

**DOI:** 10.1007/s12471-021-01620-4

**Published:** 2021-09-08

**Authors:** J. Halim, J. Brouwer, M. Lycke, M. J. Swaans, J. Van der Heyden

**Affiliations:** 1 Department of Cardiology, Sint-Jan Hospital Bruges, Bruges, Belgium; 2 Department of Cardiology, St. Antonius Hospital Nieuwegein, Nieuwegein, The Netherlands

**Keywords:** Transcatheter aortic valve replacement, Aortic stenosis, Computed tomography, Borderline annulus size, Patient-specific computer simulation

## Abstract

**Objectives:**

The aim of this study is to evaluate device size selection in patients within the borderline annulus size range undergoing transcatheter aortic valve replacement (TAVR) and to assess if pre-procedural patient-specific computer simulation will lead to the selection of a different device size than standard of care.

**Background:**

In TAVR, appropriate device sizing is imperative. In borderline annulus size cases no standardised technique for tailored device size selection is currently available. Pre-procedural patient-specific computer simulation can be used, predicting the risk for paravalvular leakage (PVL) and need for permanent pacemaker implantation (PPI).

**Methods:**

In this multicentre retrospective study, 140 patients in the borderline annulus size range were included. Hereafter, device size selection was left to the discretion of the operator. After TAVR, in 24 of the 140 patients, patient-specific computer simulation calculated the most appropriate device size expected to give the lowest risk for PVL and need for PPI. In these 24 patients, device size selection based on patient-specific computer simulation was compared with standard-of-care device size selection relying on a standardised matrix (Medtronic).

**Results:**

In a significant proportion of the 140 patients (26.4%) a different device size than recommended by the matrix was implanted. In 10 of the 24 patients (41.7%) in whom a computer simulation was performed, a different device size was recommended than by means of the matrix.

**Conclusions:**

Device size selection in patients within the borderline annulus size range is still ambiguous. In these patients, patient-specific computer simulation is feasible and can contribute to a more tailored device size selection.

## What’s new?

- Device size selection in transcatheter aortic valve replacement (TAVR) patients within the borderline annulus size range is still ambiguous and a standardised technique is lacking.
- Pre-procedural patient-specific computer simulation (FEops HEARTguide; FEops, Ghent, Belgium) can be used in TAVR to predict the risk for moderate/severe paravalvular leakage (PVL) and the occurrence of conduction disturbances.
- Pre-procedural patient-specific computer simulation can contribute to a more tailored device size selection in TAVR patients within the borderline annulus size range, potentially lowering the risk for moderate/severe PVL and the need for permanent pacemaker implantation.

## Introduction

In transcatheter aortic valve replacement (TAVR), pre-procedural planning consists of a multidetector computer tomography scan in combination with dedicated software (e.g. 3mensio, Pie Medical Imaging, Maastricht, The Netherlands) in order to select the appropriate device size [1]. In particular, the aortic annulus perimeter is an essential measurement [2, 3]. Each valve manufacturer provides a standardised matrix for device size selection, which is considered the standard of care. However, in a certain subset of patients the measurements can lead to ambiguous conclusions that can be matched by two device sizes. In this case, device size selection is left to the discretion of the operator; a possible strategy is to implant the larger device size. However, choosing the larger device size is not always the best option. Oversizing can lead to annulus rupture and conduction disturbances, while undersizing can lead to significant paravalvular leakage (PVL) [2, 4, 5]. Anticipating an increasing number of TAVR procedures in younger and low-risk patients, it becomes essential to find a standardised technique for appropriate device sizing in borderline annulus size cases to improve clinical outcomes [6, 7].

Recently, pre-procedural patient-specific computer simulation (FEops HEARTguide; FEops, Ghent, Belgium) was introduced as a potential tool for TAVR. This cloud-based technology uses acquired pre-procedural CT images to accurately predict the interaction between the implanted device and the surrounding anatomy. More specifically, simulations can be performed with different device sizes and implantation depths and subsequently the risk for PVL and need for permanent pacemaker implantation (PPI) can be predicted. Small observational studies have proven its ability to accurately predict PVL and the occurrence of conduction disturbances in TAVR patients [8–10].

We hypothesised that in TAVR, device size selection in borderline annulus size cases is ambiguous. The goal of this study is to assess the feasibility of pre-procedural patient-specific computer simulation in borderline annulus size cases and to evaluate if it will lead to a different device size selection when compared to the standard of care.

## Methods

### Study design

**Fig. 1:**
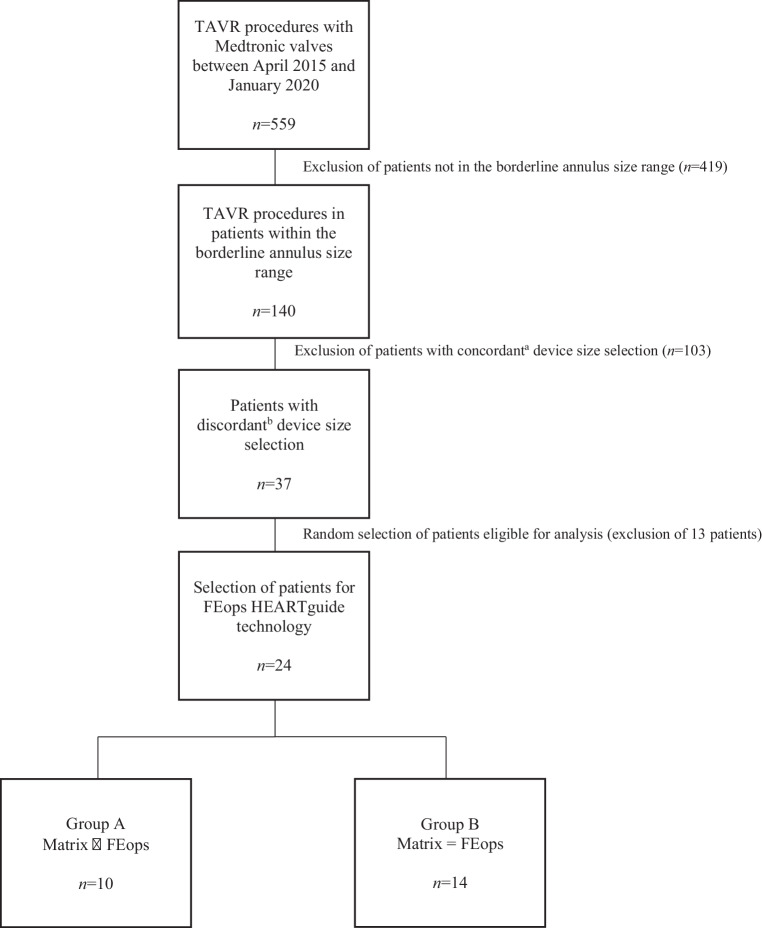
Inclusion flowchart. ^a^Device size selected as recommended by matrix, ^b^Device size used different from that recommended by the matrix Inclusion flowchart.

In this multicentre retrospective study, data from 140 patients who had undergone TAVR with a self-expanding Medtronic Evolut R or Pro valve (Medtronic, Minneapolis, MN, USA) and who fell within a borderline annulus size range based on conventional CT measurements, were collected. These 140 borderline annulus size cases were selected from a group of patients (*n* = 559) in which TAVR was performed between April 2015 and January 2020 at Sint-Jan Hospital in Bruges, Belgium or at St. Antonius Hospital in Nieuwegein, The Netherlands. All patients gave written informed consent. Then, pre-procedural CT images of 24 of the 140 patients were sent to an independent institution (FEops) and analysed by their reviewers (Fig. 1). The number of patients who underwent patient-specific computer simulation was limited due to a pre-defined financial budget. Funding was provided by FEops.

Since device sizing recommendations in the Medtronic matrix contain precise cut-off values for the annulus perimeter for each valve size and a validated borderline annulus size range is currently not available, a borderline annulus size range (i.e. grey zone) was arbitrarily determined by using a margin of 2% for each cut-off value [11].

PVL was evaluated by transthoracic echocardiogram 1 day after TAVR and was graded as: none/trace, mild, moderate or severe. Conduction disturbances were defined as the development of a high-degree atrioventricular block or a left bundle branch block.

### FEops HEARTguide technology

**Fig. 2:**
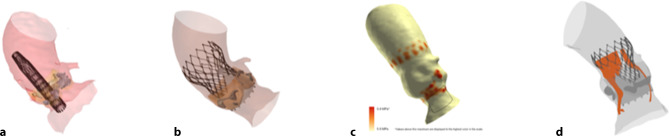
**a** FEops: three-dimensional model of the aortic root anatomy. **b** FEops: deployment of the Corevalve Evolut Pro. **c** FEops: measuring contact pressure after valve deployment. **d** FEops: predicting paravalvular leakage after valve deployment

Pre-procedural CT images were utilised to create a patient-specific three-dimensional model of the aortic root anatomy (Fig. 2). Implantation of two valve sizes and two implantation depths (high and mid-level) were then simulated. The models acquired by patient-specific computer simulation were then used to predict PVL and conduction disturbances.

Computational fluid dynamics were utilised to assess PVL severity by modelling blood flow during diastole using a fixed pressure gradient of 32 mm Hg between the aorta and the left ventricle. This fixed pressure gradient is a mean value derived from a large study population [8]. Blood flow in the left ventricle outflow tract (LVOT) was expressed in millilitres per second, whereby a value of ≥ 16.0 ml/s correlated well with ≥ moderate PVL [8]. The risk of developing conduction disturbances was predicted by measuring the exerted maximum device pressure on the area of interest (contact pressure, MPa) and the percentage of the area of interest being subjected to device pressure (contact pressure index, %). The region of the LVOT containing the atrioventricular conduction system was determined as the area of interest. A contact pressure value of > 0.39 MPa and contact pressure index of > 14% were correlated with the development of a high-degree atrioventricular block or new left bundle branch block [9].

The best-fitting device size with the ideal implantation depth could then be selected. Device size selection was based on the lowest risk for developing significant PVL and/or conduction disturbances. The FEops HEART guide reviewers were blinded to the size of the implanted valve and clinical outcomes after TAVR implantation.

### Study endpoints

The primary endpoint of this study is to assess the rate of ‘discordant’ device size selection in borderline annulus size cases. Discordant device selection is defined as the implementation of a different device size than that recommended by the matrix. Additionally, 24 patients with discordant device size selection underwent patient-specific computer simulation, after which device size selection by patient-specific computer simulation was compared to standard-of-care device size selection.

### Statistical analysis

Statistical analysis included descriptive statistics. Categorical variables are presented as counts and percentages and continuous variables as mean ± standard deviation. All analyses were conducted with SPSS v.26 (IBM, Chicago, IL, USA).

## Results

### Device size selection

Of the 140 patients, 37 (26.4%) received a valve with a different size than that recommended by the matrix (Matrix ≠ Operator). The same valve size as the one recommended by the matrix was implanted in 103 patients (73.6%) (Matrix = Operator).

### FEops analysed group

#### Baseline characteristics

In the discordant device size selection group (*n* = 37), 24 patients were randomly selected for additional patient-specific computer simulation. Baseline characteristics of the 24 patients are shown in Tab. 1. The mean age was 83.5 ± 4.3 years and 62.5% were female.

**Table 1:** Baseline characteristics

	FEops analysed group *N* = 24 *n* (%) or mean ± SD
Age, years	83.5 ± 4.3
Male	9 (37.5)
BMI	26.0 ± 3.9
RBBB	0 (0)
LBBB	3 (12.5)
Prior pacemaker	2 (8.3)
Euroscore II	5.5 ± 4.5
*Echocardiographic measurements*	
LVEF, %	57.0 ± 13.8
AV area, cm^2^	0.71 ± 0.2
AV mean gradient, mm Hg	37.7 ± 10.8
*MDCT measurements*	
Annulus perimeter, mm	74.4 ± 7.1
Perimeter derived diameter, mm	23.7 ± 2.3
Mean annulus diameter, mm	23.4 ± 2.3
Mean LVOT diameter, mm	24.1 ± 4.2
Mean STJ diameter, mm	28.1 ± 3.1
Mean SoV diameter, mm	31.1 ± 3.5
Mean SoV height, mm	23.2 ± 3.4
Maximum aorta ascendens, mm	33.0 ± 3.0

*BMI* body mass index, *RBBB* right bundle branch block, *LBBB* left bundle branch block, *LVEF* left ventricular ejection fraction, *AV* aortic valve, *MDCT* multidetector computer tomography, *LVOT* left ventricle outflow tract, *STJ* sinotubular junction, *SoV* sinus of Valsalva

#### Procedural and post-procedural data

The Evolut R system was implanted in 50% of the patients (Tab. 2). Conduction disturbances were observed in 10 patients. PPI was required in 6 patients, whereas moderate/severe PVL was present in 1 patient.

**Table 2:** Procedural data

Procedural data	FEops analysed group *N* = 24 *n* (%)
*Device system*	
Evolut R	12 (50.0)
Evolut Pro	12 (50.0)
*Implanted device size*	
23 mm	0 (0.0)
26 mm	13 (54.2)
29 mm	7 (29.2)
34 mm	4 (16.6)
Pre-dilatation	11 (45.9)
Post-dilatation	7 (29.2)

In 10 of these 24 patients (group A) the patient-specific computer simulation recommended a different valve size than the matrix (Matrix ≠ FEops). In the other 14 patients (group B) the patient-specific computer simulation recommended the same valve size as the matrix (Matrix = FEops) (Fig. 1).

### Patient-specific computer simulation (FEops analysis)

#### Paravalvular leakage

In four group‑A patients, patient-specific computer simulation concluded that the smaller device recommended by the matrix would carry a risk for moderate/severe PVL, whereas the larger device size would not involve such a risk (Tab. 3). The larger device was implanted in these four patients and did not result in moderate/severe PVL.

**Table 3:** FEops analysis

	Group A (Matrix ≠ FEops) *N* = 10 *n*	Group B (Matrix = FEops) *N* = 14 *n*
*PVL*		
Prevention of moderate/severe PVL by FEops/operators’ selection of a larger device size	4	NA
Inaccurate prediction of moderate/severe PVL by FEops	2	2
Moderate/severe PVL present after TAVR, but not predicted by FEops	0	1
Accurate prediction of no/mild PVL by FEops	4	11
*Conduction disturbances*		
Conduction disturbances and pacemaker implant which could have been prevented by high implantation	2	3
Conduction disturbances accurately predicted by FEops	2	1
Inaccurate prediction of the development of conduction disturbances	1	0
No conduction disturbances predicted or unable to analyse by FEops	5	9

*PVL* paravalvular leakage, *TAVR* transcatheter aortic valve replacement

#### Conduction disturbances

In six patients a risk for developing conduction disturbances was predicted at mid-level implantation, whereas device deployment in a high position did not involve any risk of developing conduction disturbances (Tab. 3). In five of these six patients (two patients in group A and three in group B), mid-level implantation resulted in a PPI. In the sixth patient (group B) no conduction disturbances could be seen despite mid-level implantation.

In one other patient, the risk for conduction disturbances could not be calculated. After TAVR, a PPI was required for this patient.

## Discussion

In this retrospective multicentre study, which comprised borderline annulus size cases, the operator decided to choose a different device size than recommended by the matrix in 37 of 140 patients (26.4%).

The rationale of the operator to deviate from the matrix was multifactorial, mainly driven by personal experience. The application of patient-specific computer simulation in these borderline annulus size cases was intended to predict the outcome in a reproducible and standardised manner. In this study, the theoretical application of this technology in a subgroup of 24 patients in whom the device size used was not that recommended by the matrix led to a different valve size being selected in 10 patients (41.7%) when compared to standard-of-care device size selection.

Patient-specific computer simulation has shown its potential in assessing device-host interactions in TAVR. De Jaegere et al. [8] showed that patient-specific computer simulation can accurately predict the occurrence of moderate/severe PVL in patients undergoing TAVR. Rocatello et al. [9] revealed that two simulation-based parameters (contact pressure and contact pressure index) were predictive of developing conduction abnormalities (high-degree atrioventricular block or left bundle branch block) during TAVR. This was confirmed by Dowling et al. [10] in patients with bicuspid aortic disease.

El Faquir et al. [11] concluded that device size selection in TAVR patients is more intricate and that discordance can be present between standard-of-care device sizing and device sizing based on patient-specific computer simulation. The present study has confirmed this finding in patients within the borderline annulus size range. Additionally, this study has shown that a substantial proportion of the patients undergoing TAVR should be considered a part of the borderline annulus size range group. This was the case in 25% of our TAVR patients.

We can conclude that implementation of patient-specific computer simulation is feasible in borderline annulus sizing range situations and that it can lead to a different device size selection as well as the recommendation for a specific implantation depth. In our study, a larger device size was advised on the basis of patient-specific computer simulation in four patients to prevent moderate/severe PVL. However, consistently choosing the larger device size is not always the best option taking into consideration the potential risk for annulus rupture and the need for PPI. Furthermore, it is indeed common practice to aim for high implantation to avoid pressure being exerted on the conduction system. Nevertheless, patient-specific computer simulation can provide us with information concerning in which patients device deployment in a high position is crucial to prevent the need for PPI. In our study, a PPI could have been prevented in five patients if high implantation had been used.

Thus, a more tailored approach is required during device size selection of TAVR patients considered to be in the borderline annulus size range. We believe that pre-procedural patient-specific computer simulation has the potential to play a key role in this matter. Importantly, patient-specific computer simulation is also applicable for other transcatheter heart valve systems. In our study, for practical reasons only patients in which an Evolut R/Pro valve was implanted were included. A randomised controlled trial is an essential first step to assess if device size selection by patient-specific computer simulation in patients within the borderline annulus size range will indeed lead to better clinical outcomes compared to standard-of-care device size selection. Lastly, future studies will be needed to validate and define the borderline annulus size range.

### Limitations

This study has several limitations, the first being the small sample size. Second, in this study we arbitrarily chose a 2% for each cut off value to define the borderline annulus size range. This cut-off value has not been validated. Moreover, this is an observational study in which device size selection was evaluated by two modalities. A randomised controlled trial is needed to assess whether clinical outcomes can be improved by the use of a patient-specific computer simulation.

Finally, the accuracy of the patient-specific computer simulation is susceptible to improvement: earlier published data revealed a calculated sensitivity and specificity of 0.72 and 0.78, respectively, for predicting moderate/severe PVL and 0.95 and 0.54, respectively, for predicting the development of conduction disturbances for a contact pressure index of 14%. By adding a contact pressure value of > 0.39 MPa, the accuracy of predicting conduction disturbances was increased [8–10]. These limitations of patient-specific computer simulation could be observed in our study as well.

## Conclusion

Device size selection in TAVR patients considered to be in the borderline annulus size range is still ambiguous. Our results show that patient-specific computer simulation is feasible in these cases and that it may contribute to a tailored device size selection, decreasing the risk for significant PVL and PPI need.
